# Chlorogenic acid protects against myocardial ischemia–reperfusion injury in mice by inhibiting Lnc Neat1/NLRP3 inflammasome-mediated pyroptosis

**DOI:** 10.1038/s41598-023-45017-2

**Published:** 2023-10-18

**Authors:** Xin Chai, Zhengwei Liang, Junshi Zhang, Jing Ding, Qian Zhang, Sha Lv, Yazhu Deng, Rongrui Zhang, Deqin Lu

**Affiliations:** 1 Department of Radiology, The Affiliated Hospital of Guizhou Medical University, Guiyang, 550004 China; 2 Guizhou Provincial Key Laboratory of Pathogenesis and Drug Research on Common Chronic Diseases, Guizhou Medical University, Guiyang, 550025 China; 3 Department of Pathophysiology, Guizhou Medical University, Guiyang, China

**Keywords:** Cardiology, Pathogenesis

## Abstract

Increasing evidences demonstrate that chlorogenic acid (CGA), a polyphenol with multiple effects such as anti-inflammatory and anti-oxidation, protects against myocardial ischemia–reperfusion injury (MIRI) in vitro and in vivo. But its detailed cardiac protection mechanism is still unclear. The MIRI mice model was established by ligating the left anterior descending branch (LAD) of the left coronary artery in C57BL/6 mice. Sixty C57BL/6 mice were randomly divided into four groups. CGA group and CGA + I/R group (each group *n* = 15) were gavaged with 30 mg/kg/day CGA for 4 weeks. Sham group and I/R group mice (each group *n* = 15) were administered equal volumes of saline. In vitro MIRI model was constructed by hypoxia and reoxygenation of HL-1 cardiomyocytes. The results showed that CGA pretreatment reduced myocardial infarction size and cTnT contents in serum, simultaneously reduced the levels of Lnc Neat1 expression and attenuated NLRP3 inflammasome-mediated pyroptosis in myocardial tissue. Consistent with in vivo results, the pretreatment of 0.2 μM and 2 μM CGA for 12 h in HL-1 cardiomyocytes depressed hypoxia/reoxygenation-induced Lnc Neat1 expression, NLRP3 inflammasome activation and pyroptosis. Lnc Neat1 shRNA transfection mediated by lentivirus in HL-1 cardiomyocytes significantly reduced activation of NLRP3 inflammasome and pyroptosis. Our findings suggest that CGA protects against MIRI by depressing Lnc Neat1 expression and NLRP3 inflammasome-mediated pyrotosis. Inhibiting the levels of Lnc Neat1 expression may be a therapeutic strategy for MIRI.

## Introduction

Acute myocardial infarction (AMI) has become the leading cause of death^1^. Reperfusion therapy is the most effective approach for AMI but frequently causes myocardial ischemia–reperfusion injury (MIRI) leading to myocardial stunning, reperfusion arrhythmias and adverse cardiac remodeling^2^. Numerous studies have demonstrated that the molecular mechanism underlying MIRI involves the accumulation of inf lammatory factors, the generation of reactive oxygen species (ROS), destabilization of lysosomes, potassium efflux and DNA damage^3–5^.Reperfusion therapy is the most effective approach for AMI but frequently cause myocardial ischemia–reperfusion injury (MIRI) leading to myocardial stunning and reperfusion arrhythmias, et al. Massive researches confirm that the molecular mechanism in myocardial I/R injury could be mediated through increased inflammation, reactive oxygen species (ROS) generation, lysosomal destabilization and potassium efflux^4,5^. It has been demonstrated that inflammation plays an important role in triggering and aggravating myocardial injury by inducing inflammatory programmed cell death, namely pyroptosis^6^. After myocardial ischemia reperfusion, damage-associated molecular patterns (DAMPs) injure cardiomyocytes by binding to pattern recognition receptors (PRRs), including NOD-like receptors (NLRs), Toll-like receptors (TLRs), which subsequently lead to the activation of inflammatory signaling pathways and pyroptosis^7^. Reduced inflammation has been found to be closely associated with reduced pyroptosis in MIRI^6,8^. Previous evidence has demonstrated that β-Asarone pretreatment in MIRI rats reduces the protein expression levels of ASC and NLRP3, and lower cleavage activation levels of caspase-1 and GSDMD to relieve MIRI^8^ . Therefore, depressing of pyroptosis and inflammation cascade may be an important measure in MIRI management.

Recently, the precise proinflammatory effects of NOD-like receptor (NLR) with a pyrin domain 3 (NLRP3) inflammasome have been widely studied, and its role in promoting the development of MIRI has been attracted more attention. NLRP3 is a cytosolic innate immune receptor and forms multiprotein complexes with the adaptor protein apoptosis-associated speck-like protein containing a CARD (ASC) and pro-caspase-1 after being activated^9^. Previous studies have shown that NLRP3 inflammasome activation leads to caspase-1 activation-mediated pyroptosis and inflammatory cascades^9,10^. Furthermore, during MIRI the leucine-rich repeat (LRR) domain of the NLRP3 inflammasome recognizes endogenous signals^10^, including potassium efflux, lysosomal destabilization, and mitochondrial ROS generation. After LRR domain of NLRP3 is activated, the pyrin domain (PYD) of NLRP3 homotypically interacts with that of ASC and induce the oligomerization of ASC. Then, the caspase recruitment domain (CARD) of ASC recruits pro-caspase-1^11,12^. Active caspase-1 processes pro-interleukin (IL)-1β and pro-interleukin (IL)-18 into their biologically-active mature forms. Additionally, caspase-1 activation triggers the cleavage of GSDMD, leading to the release of its N-terminal domain. Subsequently, this domain binds to the plasma membrane and forms pores, facilitating the extracellular release of mature IL-1β and IL-18^13^. In this way, the process exacerbates MIRI by promoting the inflammatory cascade and pyroptosis^14,15^. Inhibition of NLRP3 inflammasome activation has been reported to reduce MIRI^16^. Consequently, it is necessary to explore the mechanism underlying NLRP3 inflammasome activation during MIRI to provide a new strategy to combat pyroptosis and inflammatory cascades during MIRI.

Long noncoding RNA nuclear enriched abundant transcript 1 (Lnc Neat1) has been found that locates in the nucleus and maintains the structural integrity of paraspeckles^17,18^. It also has been showed that the expression levels of Lnc Neat1 increased in hypoxic or hypoxic/reoxygenation cardiomyocytes and in heart tissue of MIRI mice^19,20^. Knockdown of Lnc Neat1 in HL-1 cardiomyocytes significantly reduces the apoptosis and necrosis induced by prolonged hypoxic injury^21^. The increased Lnc Neat1 in myocardial tissue of mice with AMI upregulates the protein expression levels of caspase-3 and Bax by sponging miR-22-3p, and decreases the protein expression levels of Bcl-2 to promote apoptosis, thus aggravating myocardial injury^22^. A previous research has demonstrated that enhanced Lnc Neat1 promotes myocardial apoptosis to aggravate myocardial ischemia injury in mice, and knockdown of Lnc Neat1 protects HL-1 cardiomyocytes from hypoxia by inhibiting apoptosis^22^. Furthermore, Lnc Neat1 contributes to inducing inflammation^23,24^, and knockdown of Lnc Neat1 suppresses ASC oligomerization and caspase-1 cleavage in primary macrophages to inhibit NLRP3 inflammasome activation and inflammation^23^. In addition, hypoxia-induced Lnc Neat1 is released from paraspeckles and locates to the cytoplasm to participate in NLRP3 inflammasome assembly and activation in immortalized bone marrow-derived macrophages (iBMDMs)^24^. These findings emphasize the important role of Lnc Neatl in the activation of NLRP3 inflammasome. However, it is not completely clear whether incereased Lnc Neat1 leads to pyroptosis and inflammatory cascades through NLRP3 inflammasome activation during MIRI.

More and more studies have shown that a reasonable diet plays an important role in myocardial I/R injury and may even impact its outcome ^3,25^. Chlorogenic acid (CGA), a phenolic acid compound in the diet, has received considerable attention because of its obvious cardiovascular protective effects^26,27^. Previous studies have shown that CGA prevent against MIRI in rats due to anti-inflammatory effect^28^. CGA preconditioning protects against MIRI in rats by inhibiting the release of proinflammatory cytokines such as TNF-α and IL-6 to reduce inflammation^29^. Treatment with chlorogenic acid-phospholipid complex (CGA-PC) ameliorates MIRI by reducing levels of JNK phosphorylation, cardiac mitochondrial reactive oxygen species (mtROS) and inflammation in myocardial tissue of senescence-accelerated mouse prone 8 (SAMP8) mice^30^. Accordingly, we speculated that CGA may reduce Lnc Neat1 expression and NLRP3 inflammasome activation, thus reducing pyroptosis to protect against MIRI. Therefore, in present study, we investigated that CGA pretreatment depressed Lnc Neat1/NLRP3 inflammasome-mediated pyroptosis and inflammatory cascades to protect against MIRI.

## Methods

### Experimental animals and protocol

Experimental protocols involving animals were approved by the Animal Ethics Committee of the Guizhou Medical University, Guizhou, China (Permit Number: 2000042), and conformed to the Guide for the Care and Use of Laboratory Animals published by the US National Institutes of Health (NIH Publication No. 85-23, revised 1996) and ARRIVE guidelines. Sixty male C57BL/6 mice aged 4–6 weeks were obtained from the Experimental Animal Centre of Guizhou Medical University [Animal Certificate No. 430726210100315172] , and housed in a pathogen-free environment at 24 ± 1°C, with 50% humidity and under a 12-h light–dark cycle. All the animals were subjected to adaptive feeding for one week before the experiment.

The mice were randomly divided into four groups, namely, the sham, I/R, CGA and CGA + I/R groups, and* n* = 15 in each group. The mice in the CGA group were continuously administered 30 mg/kg/day CGA (HY-N0055, MedChemExpress) solution by gavage for 4 weeks. Similarly, the control group was administered an equivalent volume of normal saline. After gavage, the LAD coronary arteries of the mice were ligated according to the methods described by Tian^31^ on day 29 to establish the MIRI mouse model.

### MIRI mouse model

The mice were fasted from food for 12 h and from water for 4 h before the operation. The method for establishing the model is briefly described as follows. The mice were anesthetized by intraperitoneal injection of 50 mg/kg 2% sodium pentobarbital and intubated. The ventilator parameters were set as respiratory rate 80 times/min, tidal volume 0.5 ml, and the breathing ratio was 5: 4 (Taimeng, China). The fourth intercostal space over the left chest of the mouse was exposed. The left anterior descending coronary artery (LAD) was found and ligated with 8-0 suture. After 30 min of ischemia, the ligation was loosened for 1 h. After the operation, blood samples were collected from the inferior vena cava into centrifuge tubes to obtain serum. Then, the mice were sacrificed, and the heart tissues were quickly placed in liquid nitrogen or 4% paraformaldehyde for fixation.

### TTC staining

The myocardial infarct size was measured using 2,3,5-triphenyltetrazolium chloride (TTC) staining. Briefly, the hearts were harvested and washed with normal saline. The hearts were frozen at −80 °C for 10 min and then sectioned from apex to base into approximately 2–3 mm thick slices. The sections were incubated in a solution of 2% TTC (Solarbio, China) at 37 °C for 30 min in the dark and then fixed in 4% paraformaldehyde. Brick red staining indicated nonischemic areas, and pale areas of the heart were considered infarct areas. Photos were captured using a digital camera, and then, the relative infarct size was analyzed with ImageJ software (NIH, USA).

### Measurement of LDH, CK-MB and cTnT levels in serum

The blood samples were collected from the inferior vena cava. The activity of lactate dehydrogenase (LDH) and creatine kinase MB isoenzyme (CK-MB) in the mouse serum were measured by an automatic biochemical analyzer (Roche Cobas C702, Roche, Switzerland). The content of cardiac troponin T (cTnT) in the mouse serum was measured by an electroluminescent detection system (Roche Cobas 602, Roche, Switzerland).

### Hematoxylin–eosin (HE) staining

The collected heart tissues were fixed in 4% paraformaldehyde, dehydrated by an alcohol gradient, embedded in paraffin, and finally cut into sections. The sections were then deparaffinized with xylene and hydrated with gradient alcohol, followed by hematoxylin–eosin staining (Solarbio, China). After another round of dehydration, pathological changes in the myocardial tissues in the stained sections were observed under an optical microscope (Olympus, Japan).

### Immunohistochemistry (IHC) staining

Myocardium embedded in paraffin was sliced into 5 µm slices, followed by dehydration in graded alcohol and deparaffinization in dimethylbenzene. After antigen retrieval and blocking, the sections were incubated with primary antibodies against the following targets at 4 °C overnight: NLRP3 (Immunoway, YT5382), ASC (Immunoway, YT0365), cleaved-caspase-1 (Affinity, AF4005), and cleaved N-terminal GSDMD (Affinity, DF12275). The next day, added dropwise with the secondary antibody and added with a streptavidin-peroxidase solution for 10 min. Then, diaminobenzidine (DAB) was used to develop color and hematoxylin was used to reverse stain the nucleus. Finally, the sections were observed with an optical microscope (Olympus, Japan). ImageJ software (NIH, USA) was used to quantify the percentage of positive staining in IHC examinations.

### Cell culture

HL-1 cardiomyocytes were purchased from Procell (WuHan, China). HL-1 cardiomyocytes were cultured according to the manufacturer’s instructions and used for experiments at passages 4–8. HL-1 cardiomyocytes were cultured in minimum essential medium (MEM, Gibco, USA) supplemented with 10% fetal bovine serum (FBS, Biological Industries, Israel) at 37 °C in a 5% CO_2_ incubator (Thermo Fisher, USA). When the HL-1 cardiomyocytes reached 70–80% confluence, the medium was replaced with fresh medium for subsequent treatments.

### Hypoxia/reoxygenation(H/R) model establishment and CGA pretreatment of HL-1 cardiomyocytes

HL-1 cardiomyocytes were cultured in six-well plates for 12 h, and then, the medium was replaced with glucose-free, FBS-free MEM. After that, the cells were cultured in a three-gas incubator (94% N_2_ and 5% CO_2_, 37 °C, Thermo Fisher, USA) for 4 h. After 4 h of exposure to hypoxic conditions, the medium was replaced with 10% FBS MEM, and the 6-well plates were transferred into a regular incubator and incubated at 37 °C for 4 h^32^.

To verify the protective effect of CGA, HL-1 cardiomyocytes were pretreated with different concentrations of CGA (0.2 μM CGA and 2 μM CGA) for 12 h and then subjected to hypoxia for 4 h and reoxygenation for 4 h.

### RNA isolation and qRT-PCR

Total RNA was isolated from mouse heart tissues or HL-1 cardiomyocytes using TRIzol reagent (Invitrogen, USA). The RNA was reverse-transcribed into cDNA using the Hifair III 1st Strand cDNA Synthesis SuperMix kit (11141ES60, YEASEN, China). The diluted cDNA was then used for qRT-PCR using Hieff UNICON Universal Blue qPCR SYBR Green Master Mix (10 μL/well, HB200331, YEASEN, China) according to the amplification instructions. β-Actin was used as a housekeeping gene to normalize mRNA expression levels. The relative expression levels of Lnc Neat1 and the relative mRNA expression levels of NLRP3 and ASC were quantified by the 2^−ΔΔCT^ method. The primers used as followed: Lnc Neat1, 5′-GGC ACA AGT TTC ACA GGC CTA CAT GGG-3′ (forward) and 5′-GCC AGA GCT GTC CGC CCA GCG AAG-3′ (reverse); NLRP3, 5′-AGA AGA GAC CAC GGC AGA AG-3′ (forward) and 5′-CCT TGG ACC AGG TTC AGT GT-3′ (reverse); ASC, 5′-CTG ACG GAT GAG CAG TAC CA-3′ (forward) and 5′-AGT CCT TGC AGG TCC AGT TC-3′ (reverse); and β-actin, 5′-GTA GCC ATC CAG GCT GTG TT-3′ (forward) and 5′-ATG TCA CGC ACG ATT TCC CT-3′ (reverse).

### Western blotting analysis

Heart tissues or HL-1 cardiomyocytes were homogenized in RIPA lysis buffer and centrifuged at 12,000*g* at 4 °C for 25 min. Then, protein supernatants were obtained and added with loading buffer to boil for 10 min. A total of 10 to 30 µg proteins were used to perform western blot as previously reported^33^. Primary antibodies for NLRP3 (CST, 15101), pro-caspase-1 (CST, 24232), cleaved-caspase-1 (CST, 89332), GSDMD (Abcam, ab219800), cleaved N-terminal GSDMD (CST, 10137), IL-1β (Bioworld, BS6067), IL-18 (Bioworld, BS6823) were used, respectively. β-tubulin was used as an internal reference for protein analysis.

### ASC oligomerization preparation

HL-1 cardiomyocytes were collected after the indicated treatments, resuspended in ice-cold buffer and lysed by shearing 10 times through a 27-gauge syringe according to the previous study^24^. Cell lysates were centrifuged at 520*g* for 8 min at 4 °C. The supernatant cross-linked by incubation with 2 mM disuccinimidyl suberate (DSS, APExBIO) for 30 min at room temperature. The samples were analyzed by Western blotting with an anti-ASC antibody (Bioworld, BS6205).

### Propidium iodide (PI) staining

The integrity of HL-1 cardiomyocyte membranes were observed by PI staining. After stimulation, the cells were washed 3 times with 1× PBS. Then, 5 µL PI (Bioworld, China) was mixed with 1× assay buffer, and each well was stained at 37 °C for 15 min in the dark. Fluorescence microscopy (Olympus, Japan) was used to acquire images of the cells immediately after staining. The percentage of positive cells was assessed with ImageJ (NIH, USA).

### Lentiviral vector infection

The pLV-U6-Neat1shRNA-GFP-Puro, psPAX2 and pMD2.G plasmids were constructed by Hejin Biotechnology Co., LTD. (Guizhou, China). Lentiviral recombinant plasmids (4.4 μg) was cotransfected together with the pMD2.G (2.225 μg) and psPAX2 (3.325 μg) plasmids into HEK-293 T cells using Lipofectamine 2000 (Invitrogen, USA) in 10-cm dishes. The viral supernatant was collected after transfection for 48–72 h. The supernatant was centrifuged (2000 rpm for 10 min at 4 °C), filtered through 0.45 μm filters, and then placed onto HL-1 cardiomyocytes supplemented with 8 ng/μL polybrene (Solarbio, China). After 48 h, the efficiency of infection was evaluated using a fluorescence microscope.

### Statistical analysis

The data are expressed as the mean ± SD. Differences between two groups were analyzed by Student’s two-tailed t test, and differences between more than two groups were analyzed by one-way ANOVA using SPSS 29.0 software (IL, USA). *P* < 0.05 was considered statistically significant.

## Results

### CGA pretreatment reduces the myocardial infarct size and myocardial injury markers in MIRI mice

**Figure 1:**
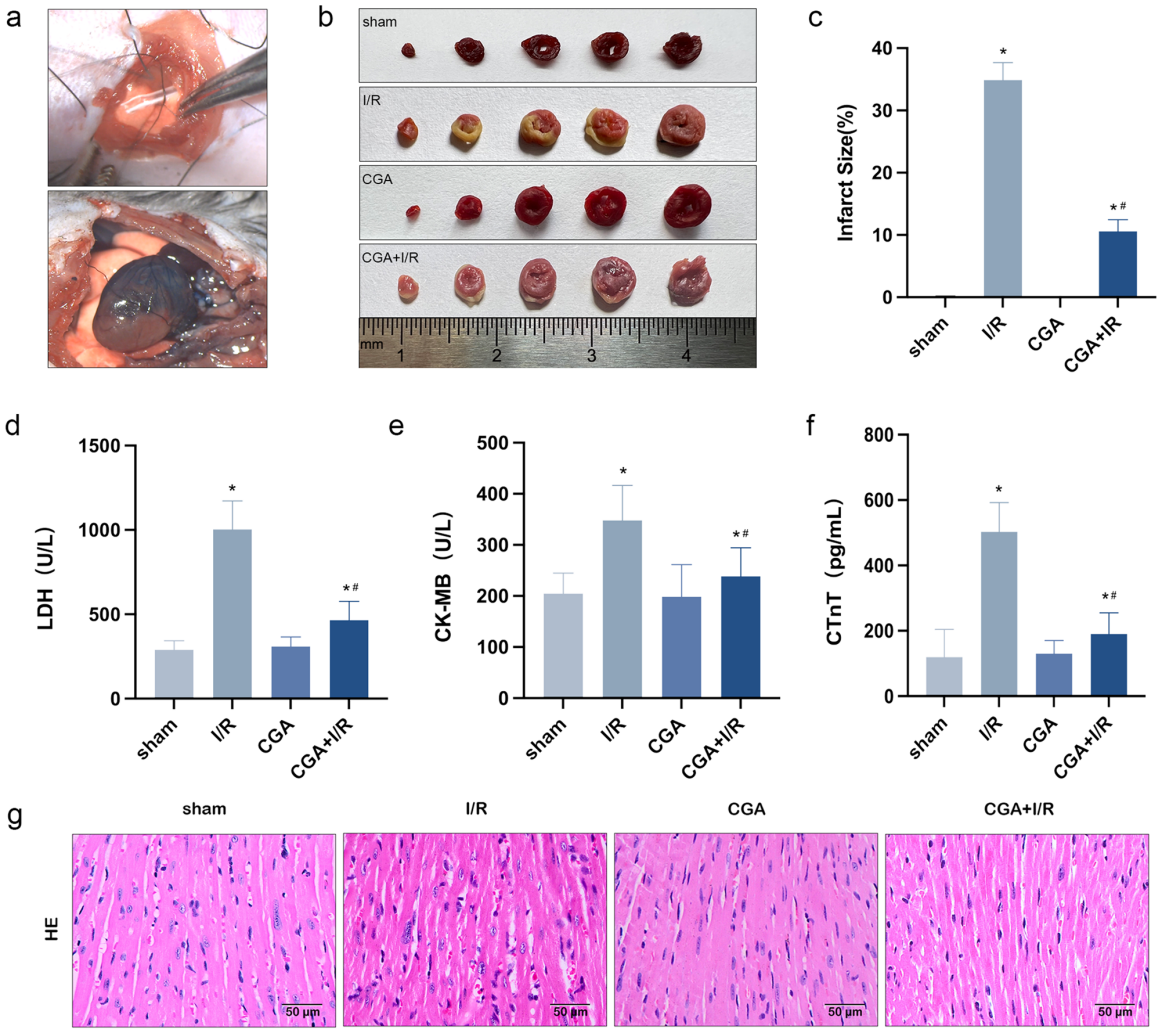
The protective effect of CGA pretreatment on MIRI in mice. (**a**) Evans blue was injected via the aorta after myocardial ischemia reperfusion. Blue area: noninfarcted area, red area: infarcted area. (**b,c**) The proportion of the infarct area in the CGA + I/R group was far lower than that in the I/R group, and the infarcted tissue (white area) was identified by TTC staining. (**d,e**) The effect of CGA on LDH and CK-MB activity in I/R-injured mice. The activity of LDH and CK-MB in mouse serum were measured by an automatic biochemical analyzer system. (**f**) The effect of CGA on content of cTnT in I/R-injured mice. The content of cTnT in mouse serum was measured by an automatic biochemical analyzer and electroluminescence detection system. (**g**) Pathological changes of myocardial tissue were observed by HE staining. Representative images were captured using an optical microscope (×400  magnification). The data are expressed as the mean ± SD. *n* = 15 mice in each group. *P < 0.05 vs. the sham group, ^**#**^*P* < 0.05 vs. the I/R group.

In the present study, the MIRI model was established by ligating the LAD of C57BL/6 mice, and this model was used to verify the protective effect of CGA against MIRI. Figure 1a shows the surgical area of myocardial infarction and the ligation site in the model. After 30 min of ischemia and 1 h of reperfusion of the LAD, evans blue was injected through the aorta, and evans blue staining allowed the visualization of infarcted tissues (red area) and uninfarcted tissues (blue area); the results revealed obvious ischemic symptoms. As shown in Fig. 1b, TTC staining revealed a white area of myocardial infarction. According to statistical analysis (Fig. 1c), the myocardial infarction size of mice in the I/R group was approximately 34.9 ± 2.3% and that of mice in the CGA + I/R group was approximately 10.6 ± 1.5%; these results showed a statistically significant difference between the two groups (*P* < 0.05) and suggested that CGA pretreatment could significantly reduce myocardial infarction caused by I/R injury. Similarly, compared with the sham group, the I/R group had significantly increased myocardial enzyme LDH, CK-MB activity and myocardial injury index cTnT content in the serum (*P* < 0.05), and CGA pretreatment significantly reduced the LDH and CK-MB activity and the cTnT content in the serum of I/R model mice (*P* < 0.05), as shown in Fig. 1d–f. Histopathological changes were evaluated by HE staining. As shown in Fig. 1g, mice in the sham group had heart tissue with normal morphology. The changes observed in the infarct area of the I/R group included disordered arrangement of myocardial fibers, myocardial edema, nuclear fragmentation and inflammatory infiltration. While myocardial edema and inflammatory infiltration were lower in CGA + I/R group than in I/R group. In addition, no obvious nuclear fragmentation was observed in the CGA + I/R group, and the morphology was more normal. These data suggest that CGA pretreatment has a protective effect on myocardial tissues in MIRI model mice.

### CGA protects against MIRI by reducing Lnc Neat1 expression levels and suppressing NLRP3 inflammasome activation in mice

**Figure 2:**
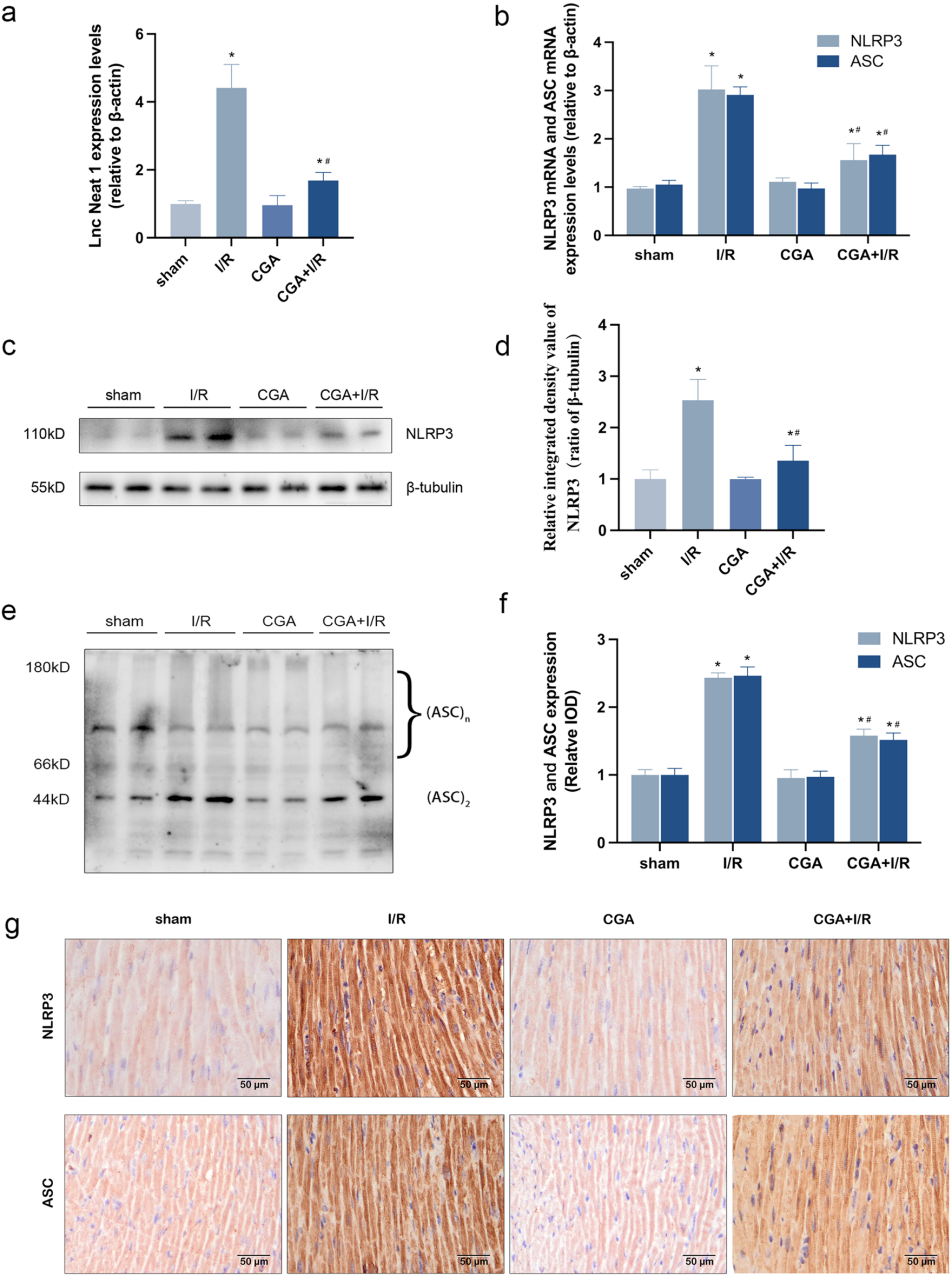
CGA pretreatment decreases the expression levels of Lnc Neat1 and inhibits the activation of the NLRP3 inflammasome in MIRI mice. (**a**) CGA pretreatment reduced the I/R injury-induced expression levels of Lnc Neat1 in myocardial tissue.The expression levels of Lnc Neat1 were measured by qRT-PCR. (**b**) CGA pretreatment decreased the I/R injury-induced mRNA expression levels of NLRP3 and ASC. The mRNA expression levels of NLRP3 and ASC were measured by qRT-PCR. (**c,d**) CGA pretreatment lowered the I/R injury-induced protein expression levels of NLRP3 in myocardial tissue. The protein expression levels of NLRP3 were measured by Western blotting analysis. (**e**) CGA pretreatment markedly inhibited I/R injury-mediated ASC oligomerization in mouse myocardial tissue. ASC oligomerization was measuring by Western blotting analysis. (**f,g**) CGA pretreatment decreased the I/R injury-induced protein expression of NLRP3 and ASC in the cytoplasm of cardiomyocytes. Immunohistochemistry was used to measure the protein expression levels of NLRP3 and ASC in the cytoplasm of cardiomyocytes. Representative images were captured using an optical microscope (×400  magnification). The data are expressed as the mean ± SD. *n* = 15 mice in each group. The full-length blots are presented in Supplementary Fig. 1. *P < 0.05 vs. the sham group, ^**#**^*P* < 0.05 vs. the I/R group.

The qRT-PCR results showed that compared with those in the sham group, the expression levels of Lnc Neat1 in the myocardial tissues of mice in the I/R group were significantly increased. As expected, pretreatment with CGA significantly reduced the I/R damage-mediated high expression levels of Lnc Neat1 (*P* < 0.05), as shown in Fig. 2a. The results suggest that Lnc Neat1, which is upregulated in the myocardium of MIRI model mice, may be an important factor promoting myocardial injury. Besides, CGA significantly downregulated the mRNA expression levels of NLRP3 and ASC in myocardial tissues of mice which were stimulated by I/R (*P* < 0.05) , as displayed in Fig. 2b. Western blotting assays showed that NLRP3 protein expression levels were upregulated (*P* < 0.05) and ASC oligomerization was increased in the myocardial tissues of MIRI model mice. However, an opposite expression pattern was observed in the I/R group with CGA pretreatment (Fig. 2c–e). Immunohistochemistry showed that the NLRP3 and ASC proteins were mainly located in the cytoplasm of cardiomyocytes in mice. The high protein expression levels of NLRP3 and ASC were observed in myocardial tissues of I/R group (*P* < 0.05), while CGA pretreatment reversed the effect of I/R injury (Fig. 2f, g). Based on these findings, CGA considerably lowered the mRNA and protein expression levels of NLRP3 and ASC, thus inhibited the activation of the NLRP3 inflammasome after I/R injury in vivo.

### CGA protects HL-1 cardiomyocytes from H/R injury by decreasing the expression levels of Lnc Neat 1 and inhibiting the activation of the NLRP3 inflammasome

**Figure 3:**
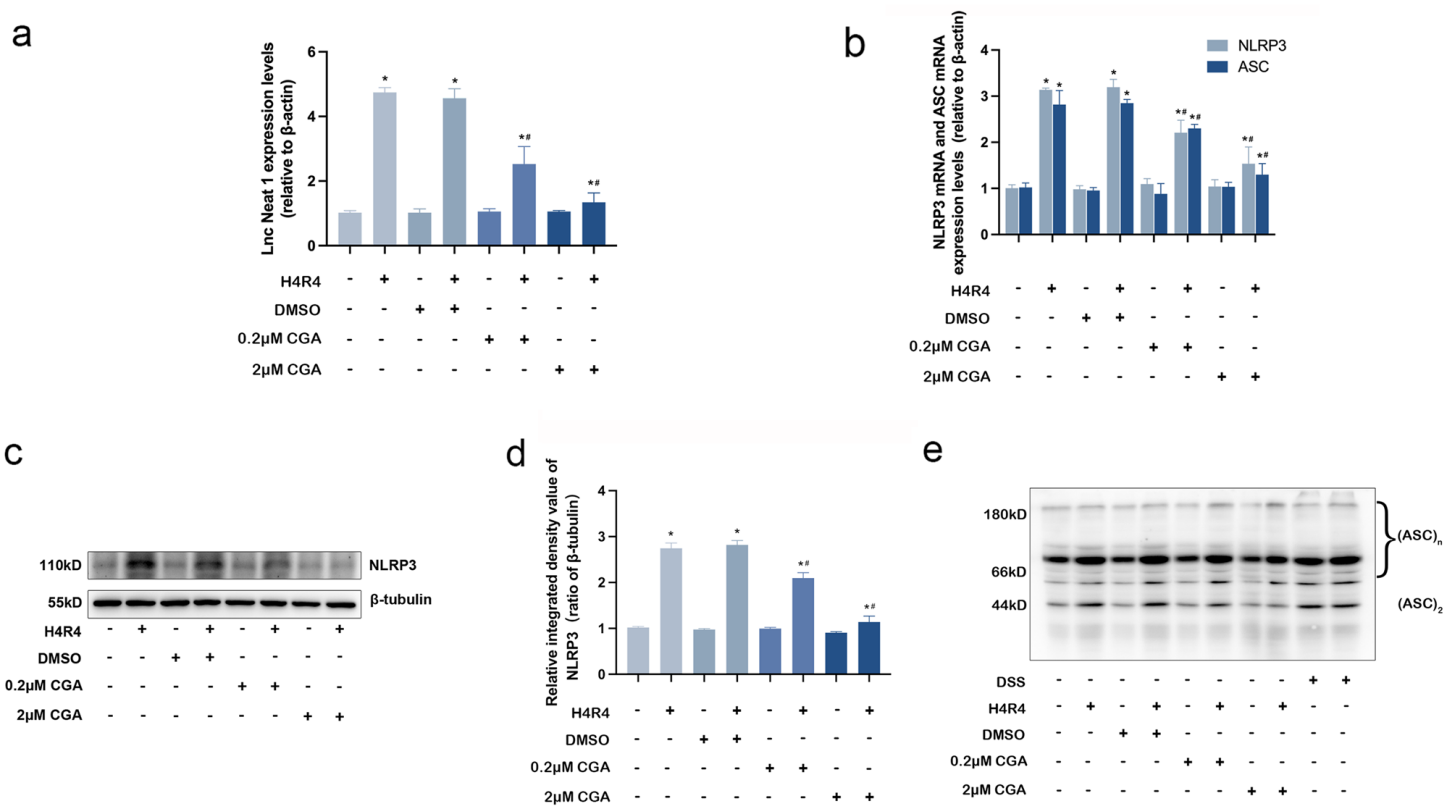
CGA pretreatment reduces the expression levels of Lnc Neat1 and inhibits the activation of the NLRP3 inflammasome in HL-1 cardiomyocytes exposed to H/R. (**a**) HL-1 cardiomyocytes were pretreated with 0.2 μM CGA or 2 μM CGA for 12 h before H4R4. Effect of CGA on expression levels of Lnc Neat1 in HL-1 cardiomyocytes. The expression levels of Lnc Neat1 were measured by qRT-PCR. (**b**) Effect of CGA on mRNA expression levels of NLRP3 and ASC in HL-1 cardiomyocytes. The mRNA expression levels of NLRP3 and ASC were measured by qRT-PCR. (**c,d**) CGA pretreatment reduced the H/R injury-induced high protein expression levels of NLRP3 in HL-1 cardiomyocytes. The protein expression levels of NLRP3 were measuring by Western blotting analysis. (**e**) CGA pretreatment markedly inhibited H/R injury-mediated ASC oligomerization in HL-1 cardiomyocytes. ASC oligomerization was measuring by Western blotting analysis. The data are expressed as the mean ± SD. *n* = 3 independent experiments. The full-length blots are presented in Supplementary Fig. 2. *P < 0.05 vs. the control group, ^#^ *P* < 0.05 vs. the H4R4 group.

To further verify that CGA could reduce the expression levels of Lnc Neat1 and inhibit the activation of the NLRP3 inflammasome by H/R injury, HL-1 cardiomyocytes were pretreated with 0.2 μM CGA or 2 μM CGA for 12 h before being subjected to H/R condition. The qRT-PCR results showed that, compared with the control group, the expression levels of Lnc Neat1 were upregulated by H/R injury (*P* < 0.05). Not unexpectedly, pretreatment with CGA restrained the effect by H/R-injured in HL-1 cardiomyocytes (Fig. 3a). The results suggest that Lnc Neat1, which is upregulated in HL-1 cardiomyocytes with H/R injury, was considered as a possible important factor in promoting myocardial injury. To explore the effect of CGA on the activation of NLRP3 inflammasome mediated by H/R injury in HL-1 cardiomyocytes, the mRNA expression levels of NLRP3 and ASC were detected by qRT-PCR, and the protein expression levels of NLRP3 and ASC oligomerization were detected by Western blot. H/R injury increased the mRNA expression levels of NLRP3 and ASC in HL-1 cardiomyocytes (*P* < 0.05). However, CGA pretreatment significantly reversed the effect of I/R injury, as shown in Fig. 3b. Similarly, the protein expression levels of NLRP3 and ASC oligomerization were also significantly lower in the CGA + I/R group compared with those in the I/R group (Fig. 3c–e).

### CGA protects against MIRI by inhibition of pyroptosis in vivo and in vitro

**Figure 4:**
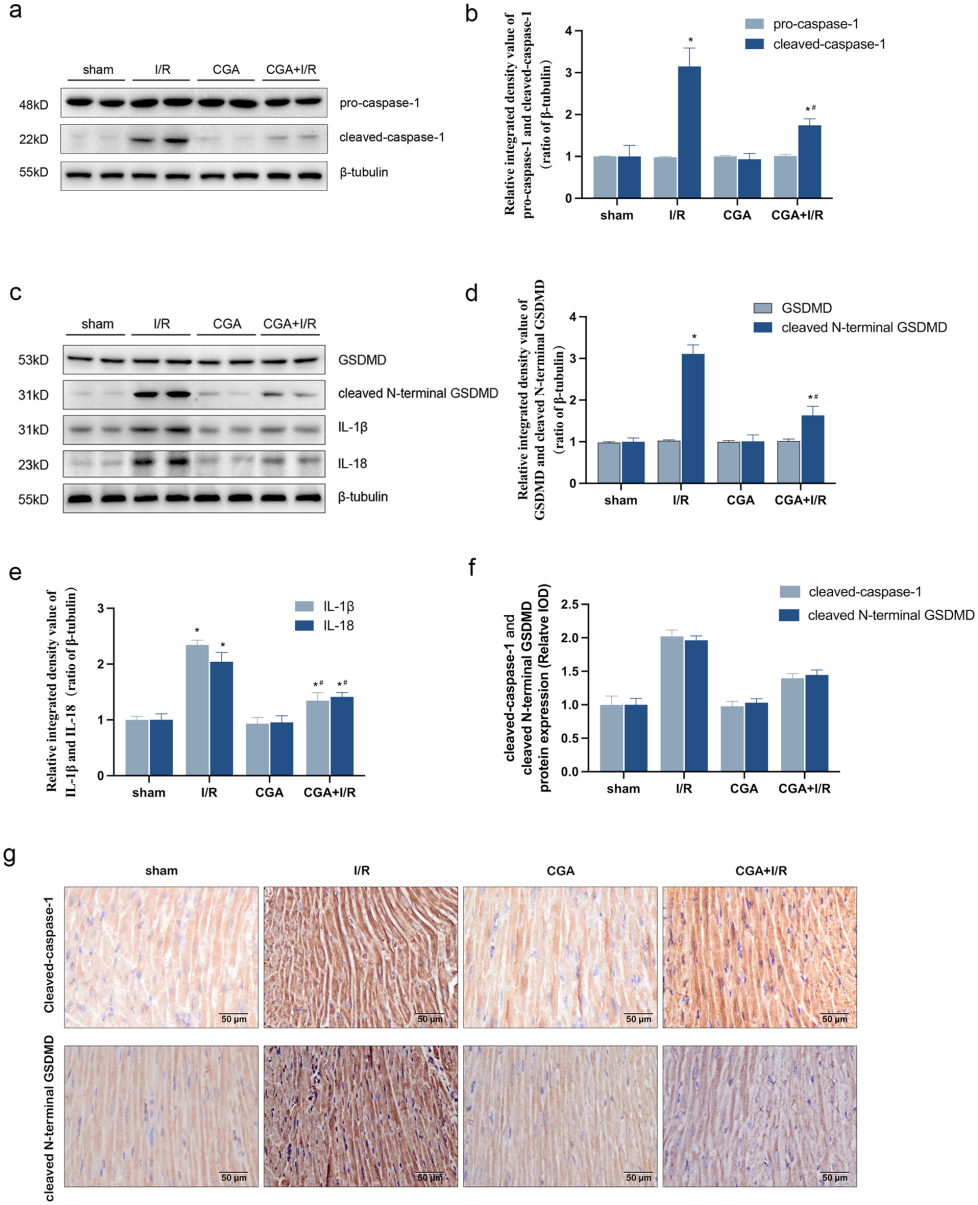
CGA pretreatment protects against MIRI in mice by inhibiting pyroptosis. (**a,b**) Effect of CGA on protein expression levels of pro-caspase-1 and cleaved-caspase-1 in I/R-injured mice.The protein expression levels of pro-caspase-1 and cleaved-caspase-1 were measuring by Western blotting analysis. (**c,e**) Effect of CGA on protein expression levels of GSDMD, cleaved N-terminal GSDMD, IL-1β and IL-18 in I/R-injured mice. The protein expression levels of GSDMD, cleaved N-terminal GSDMD, IL-1β and IL-18 were measuring by Western blotting analysis. (**f,g**) CGA pretreatment decreased the I/R injury-induced protein expression levels of cleaved-caspase-1 in the cytoplasm of cardiomyocytes. Immunohistochemistry was used to measure the protein expression levels of cleaved-caspase-1 and cleaved N-terminal GSDMD in the cytoplasm of cardiomyocytes. Representative images were captured using an optical microscope (×400  magnification). The data are expressed as the mean ± SD. *n* = 15 mice in each group. The full-length blots are presented in Supplementary Fig. 3. *P < 0.05 vs. the sham group, ^#^*P* < 0.05 vs. the I/R group.

**Figure 5:**
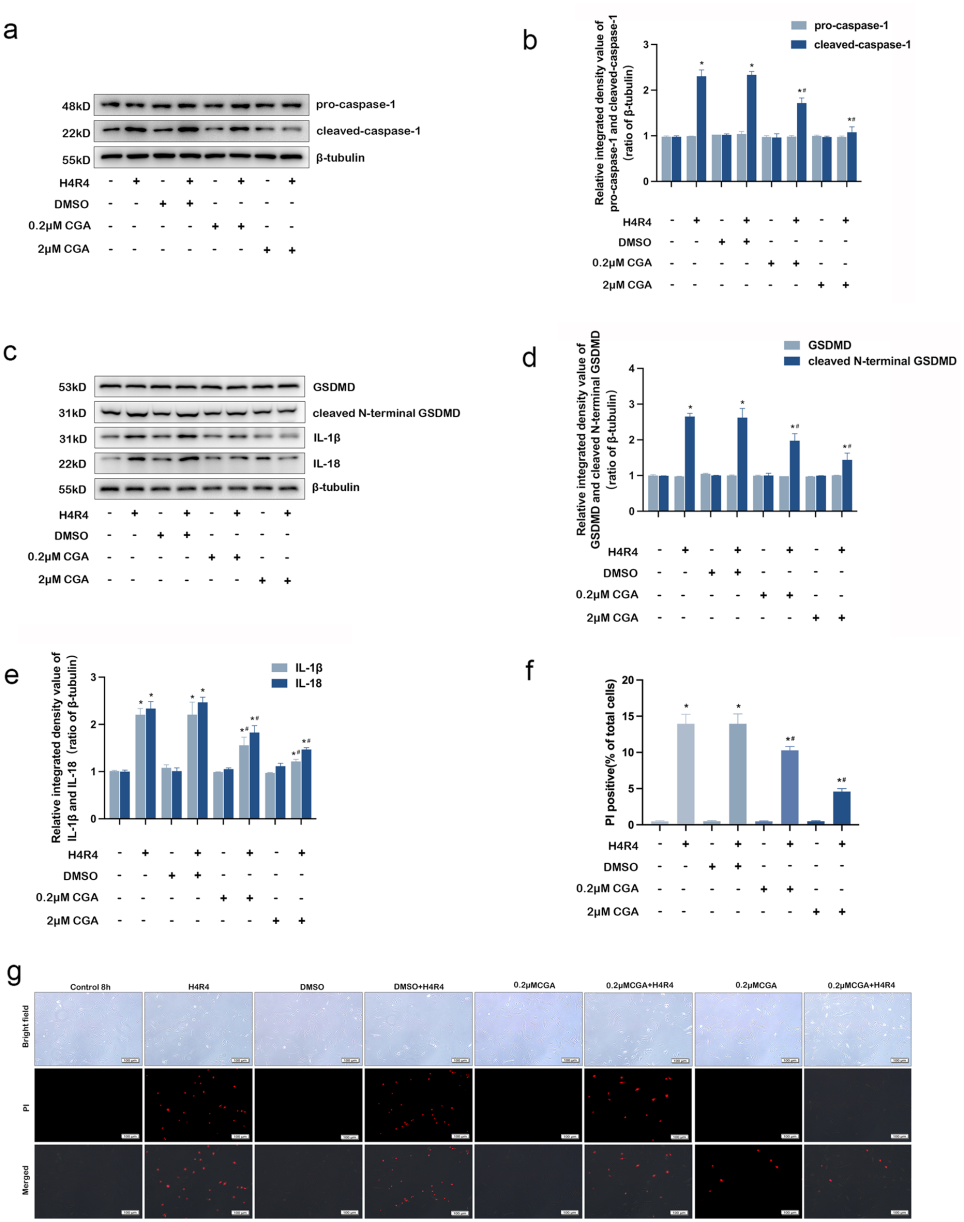
CGA pretreatment protects HL-1 cardiomyocytes against H/R injury by inhibiting pyroptosis. (**a,b**) HL-1 cardiomyocytes were pretreated with 0.2 μM CGA or 2 μM CGA for 12 h before H4R4. Effect of CGA on the protein expression levels of pro-caspase-1 and cleaved-caspase-1 in HL-1 cardiomyocytes. The protein expression levels of pro-caspase-1 and cleaved-caspase-1 were measuring by Western blotting analysis. (**c–e**) Effect of CGA on the protein expression levels of GSDMD, N-terminal GSDMD, IL-1β and IL-18 in HL-1 cardiomyocytes. The protein expression levels of GSDMD, cleaved N-terminal GSDMD, IL-1β and IL-18 were measuring by Western blotting analysis. (**f,g**) CGA pretreatment decreased the H/R injury-induced rate of positive PI staining in HL-1 cardiomyocytes. Representative images were captured using a fluorescence microscope (×100  magnification). The data are expressed as the mean ± SD. *n* = 3 independent experiments. The full-length blots are presented in Supplementary Fig. 4. *P < 0.05 vs. the control group, ^#^*P* < 0.05 vs. the H4R4 group.

To evaluate the effect of CGA on pyroptosis, pyroptosis-related proteins were detected by western blotting and immunohistochemistry. As shown in Figs. 4a–d and 5a–d, western blotting analysis showed that the protein expression levels of cleaved-caspase-1 and cleaved N-terminal GSDMD were significantly upregulated in the myocardial tissues of MIRI model mice and HL-1 cardiomyocytes by I/R injury, which was consistent with immunohistochemical results (Fig. 4f, g). However, pretreatment with CGA significantly reversed these effects (*P* < 0.05). Besides, there was no significant difference in the protein expression levels of pro-caspase-1 and GSDMD between the different groups. Moreover, the protein expression levels of IL-1β and IL-18 were significantly increased after I/R injury in vitro and in vivo (*P* < 0.05). This suggests that activation of caspase-1 cleaves pro-IL-1β and pro-IL-18 into active forms, thus promoting an inflammatory cascade. Additionally, after CGA pretreatment, protein expressions levels of IL-1β and IL-18 decreased significantly (*P* < 0.05), as shown in Figs. 4c, e and 5c, e. The cell membrane integrity was detected by PI stain. We observed that the increase of the rate of positive PI staining in HL-1 cardiomyocytes in the H/R group was remarkable (*P* < 0.05), but that in the 0.2 μM or 2 μM CGA + H/R group was not obvious (Fig. 5f, g). Moreover, 2 μM CGA pretreatment showed more significant protection against H/R injury.

### Lnc Neat1 mediates NLRP3 inflammasome activition during H/R injury

**Figure 6:**
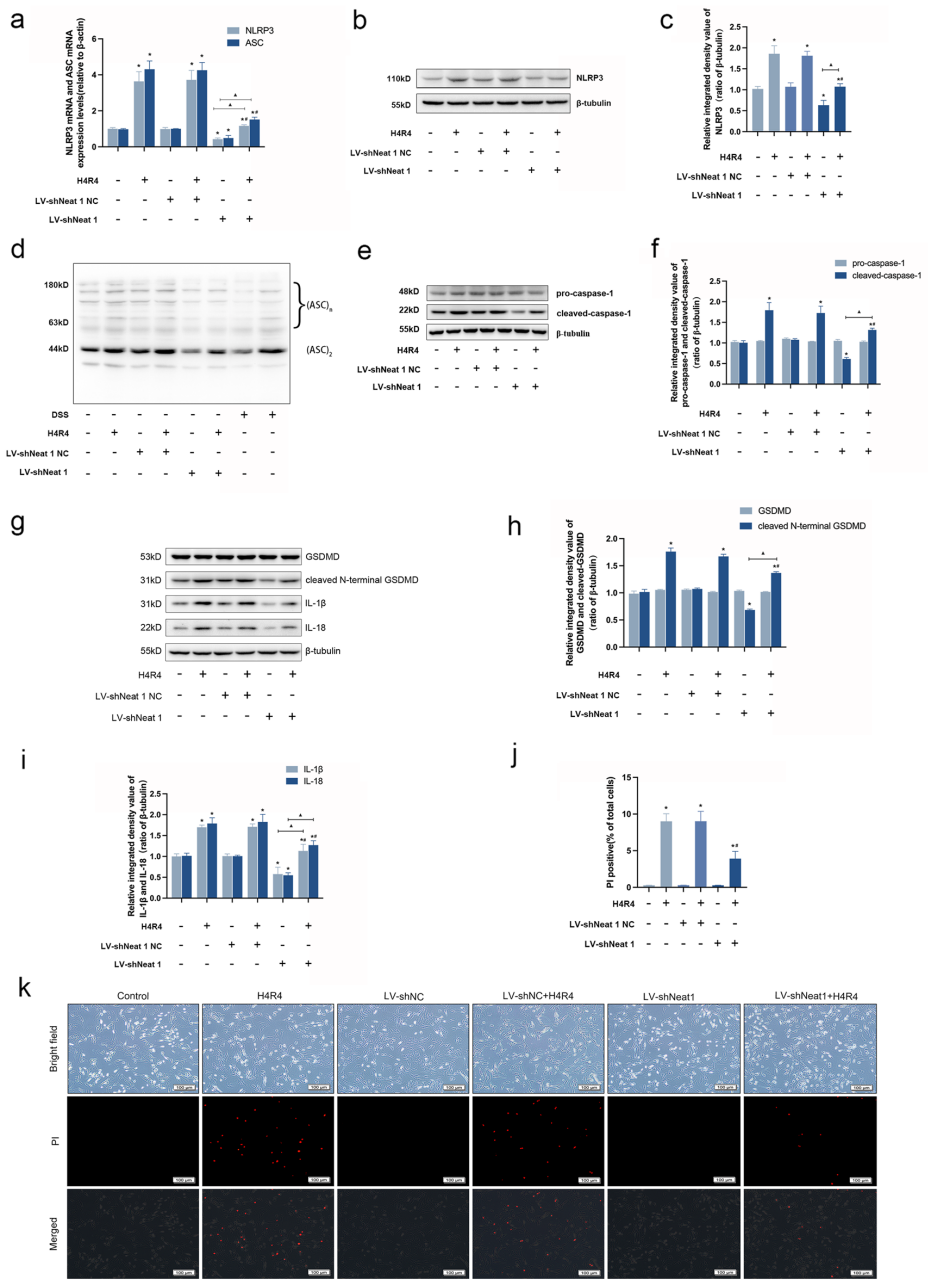
Inhibition of Lnc Neat1 reduces NLRP3 inflammasome activation and pyroptosis induced by H/R injury in HL-1 cardiomyocytes. (**a**) Effect of knocking down Lnc Neat1 on the mRNA expression levels of NLRP3 and ASC in HL-1 cardiomyocytes. The mRNA expression levels of NLRP3 and ASC were detected by qRT-PCR analysis. (**b–d**) Silencing Lnc Neat1 reversed H/R injury-induced the protein expression levels of NLRP3 and ASC oligomerization. (**e,f**) Effect of knocking down Lnc Neat1 on the protein expression levels of pro-caspase-1 and cleaved-caspase-1 in HL-1 cardiomyocytes. The protein expression levels of pro-caspase-1 and cleaved-caspase-1 were detected by Western blot analysis. (**g–i**) Knockdown of Lnc Neat1 expression attenuated the protein expression levels of cleaved N-terminal GSDMD, IL-1β and IL-18 after H/R. The protein expression levels of GSDMD、cleaved N-terminal GSDMD、IL-1β and IL-18 were detected by Western blot analysis. (**j,k**) Knocking down Lnc Neat1 expression reduced the positive rate of PI staining in HL-1 cardiomyocytes subjected to H/R injury. Representative images were captured using a fluorescence microscope (100 × magnification). Data are expressed as the mean ± SD. *n* = 3 independent experiments. The full-length blots are presented in Supplementary Fig. 5. *P < 0.05 vs. contorl group, ^#^*P* < 0.05 vs. H4R4 group, ^▲^*P* < 0.05.

To further dissect the underlying mechanism of Lnc Neat1 in H/R injury-induced NLRP3 inflammasome activation, we selected the lentivirus-mediated method to infect HL-1 cardiomyocytes after lentiviral packaging of the target plasmid in order to increase transfection efficiency and obtain cell lines with long-term stable interference with Lnc Neat1. Interestingly, the knockdown of Lnc Neat1 resulted in a strong reduction in mRNA expression of NLRP3 and ASC, an obvious downregulation in protein expression of NLRP3, and a clear decrease in oligomerization of ASC, indicative of inhibition assembly of NLRP3 inflammasome (Fig. 6a–d). In addition, this was accompanied by a noticeable reduction in the expression of pyroptosis-related proteins, such as cleaved-caspase-1, cleaved N-terminal GSDMD, IL-1β and IL-18 (Fig. 6e–i). Similarly, Knocking down Lnc Neat1 expression reduced the positive rate of PI staining in HL-1 cardiomyocytes subjected to H/R injury(*P* < 0.05)(Fig. 6j, k).

## Discussion

The present study suggests that increased Lnc Neat1 in cardiomyocytes promotes NLRP3 inflammasome-induced pyroptosis during MIRI. The data also demonstrate that CGA protects against MIRI by decreasing Lnc Neat1 expression then attenuating NLRP3 inflammasome-mediated pyroptosis and inflammatory cascades. CGA could become a novel intervention for MIRI.

Cell death is a critical event in MIRI. It has been confirmed that apoptosis, necroptosis, ferroptosis and pyroptosisplay significant roles in MIRI. Apoptosis is present in all life stages in multicellular organisms and allows for the timely removal of senescent, damaged, redundant, harmful cells, maintaining the organism’s stability^34^. Studies have shown that deletion of p53 in mice decreases cardiac injury by inhibiting apoptosis through attenuation of oxidative stress and calpain activation during ischemia–reperfusion^35^. With the deepening of research, it was found that cell death factors such as tumor necrosis factor-α could cause a kind of “programmed” necrosis, namely necroptosis^36^. Necroptosis, a form of cell death similar to the necrotic phenotype, has been identified in cardiac pathology. Recently, Song et al. showed that receptor interacting serine threonine kinase 3 is rapidly increased in hypoxia/reoxygenation-treated cardiomyocytes and is associated with myocyte necroptosis^37^. Ferroptosis is a non-apoptotic form of cell death that depends on the accumulation of intracellular iron, leading to elevated levels of toxic lipid peroxides^38^. It has been reported that cyclosporine A-loaded apoferritin inhibited ferroptosis of ischemic cardiomyocytes by increasing the protein expression of glutathione peroxidase 4 and reducing the content of lipid peroxides^39^. Pyroptosis is a kind of pro-inflammatory cell death. Compared with autophagy and apoptosis, pyroptosis has unique characteristics in morphology and mechanism^40^. Specifically, pyroptosis is a kind of cell lysis mediated by the Gasdermin family, releases inflammatory cytokines IL-1β and IL-18. A large number of studies have proved that pyroptosis is closely related to MIRI. During an MIRI event, the damage is initially caused by rapid accumulation of intracellular sodium and hydrogen, which induces adenosine triphosphate (ATP) depletion, potassium efflux, and mitochondrial ROS generation^41^. The injured cardiac parenchymal cells, damaged extracellular matrix, and released substancesact as DAMPs to activate the inflammatory response^41^. Increasing evidence indicated that MIRI was accompanied by inflammatory responses that lead to pyroptosis and promote myocardial damage^42^. In such cases, reduction in inflammatory response represents a potential target for prevention of MIRI^41–43^.

Phenolic acids are a group of chemical compounds that can be found in a variety of foods and beverages. These compounds have been proven to provide numerous health benefits and play a significant role in our daily diet. Chlorogenic acid (CGA), which belongs to the phenolic acid compound, has been extensively studied and shown to possess anti-inflammatory, antioxidant, and other biological activities^27^. Animal studies have also demonstrated that CGA can help prevent cardiovascular diseases^44,45^. Therefore, the aim of this study is to investigate the preventive effect of CGA on MIRI using a mouse model of myocardial infarction induced by ligation of the LAD coronary artery. The results of this study revealed that pretreatment with CGA led to a reduction in the size of myocardial infarction, as well as decreased levels of LDH, CK-MB activity, and the content of serum cTnT (Fig. 1d–f). Furthermore, HE staining demonstrated a decrease in myocardial edema and inflammatory response in the CGA + I/R group compared to the I/R group (Fig. 1e). These findings are consistent with previous studies^28^, suggesting that preconditioning with CGA has a protective effect on MIRI. However, further research is needed to elucidate the specific mechanisms by which CGA alleviates the inflammatory response in MIRI.

Studies have shown that NLRP3 inflammasomes exert an injurious effect in promoting pyroptosis and inflammatory cascades during myocardial ischemia–reperfusion^46^. During reperfusion, the release of large amounts of ATP with blood and oxygen reflux stimulates NLRP3 inflammasome activation, which is mediated by P2X purinoreceptor 7 (P2X7); then, activated NLRP3 inflammasomes promote caspase-1 cleavage, which induces pyroptosis and the maturation of pro-inflammatory cytokines, further exacerbating the inflammatory response and aggravating myocardial injury^47^. The NLRP3 inflammasome is a key factor in the induction of this process and is currently the most widely studied inflammasome^48,49^. Peng et al.^50^ established a mouse MIRI model by ligating the LAD and found that MIRI activates the NLRP3 inflammasome and increases the protein expression of cleaved-caspase-1, cleaved N-terminal GSDMD and IL-1β. These phenomena lead to pyroptosis and inflammatory cascades, which aggravate MIRI. Consistent with the report by Peng et al., we observed that the increased mRNA and protein expression levels of NLRP3 and ASC in myocardial tissues of MIRI model mice and HL-1 cardiomyocytes of H/R injury, which led to activated NLRP3 inflammasome (Fig. 2b–g); then, activated NLRP3 inflammasome induced pyroptosis (Figs. 3, 5). Previous studies have indicated that CGA could effectively protect against cardiovascular disease and reduce inflammation during MIRI^28,30^. However, it is still unclear whether CGA could prevent MIRI by inhibiting the activation of the NLRP3 inflammasome and subsequently reducing pyroptosis. Additionally, experiments conducted in vitro and in vivo demonstrated that CGA pretreatment significantly decreased the mRNA and protein expression levels of NLRP3 and ASC, thereby inhibiting the activation of the NLRP3 inflammasome (Figs. 2, 4). Furthermore, CGA pretreatment suppressed pyroptosis and reduced the production of pro-inflammatory cytokines IL-1β and IL-18, which were associated with NLRP3 inflammasome activation (Figs. 3, 5). These findings collectively suggested that CGA may prevent MIRI through the activation of the NLRP3 inflammasome, thus reducing pyroptosis and inflammation.

Proper control of the NLRP3 inflammasome assembly permits effective protection against MIRI while mitigating tissue damage. However, how NLRP3 inflammasome assembled remains defined. The long noncoding RNAs (lncRNAs) recognized as essential regulators of gene expression has attracted increasing attention. Lnc Neat1 was identified in a 2007 study of the enrichment of noncoding RNAs in mammalian nuclei^51^, and it is mainly located in the nuclear substructures called paraspeckles^52^. Previous studies have shown that Lnc Neat1 is a pivotal contributor to promoting MIRI and associated with the reduce of myocardial infarct size^53,54^. From the results of both in vitro and in vivo study, we observed that the expression levels of Lnc Neat1 in myocardial tissue cells were significantly increased during MIRI (Figs. 2a and 3a). In addition, we further revealed that CGA pretreatment could reduce the expression levels of Lnc Neat1 in the IRI hearts and H/R HL-1 cardiomyocytes. We therefore hypothesize that CGA has a protective effect on MIRI in mice by inhibiting Lnc Neat1. Remarkably, numerous studies have revealed that Lnc Neat1 plays a critical role in activation of NLRP3 inflammasome. In the study by Yao et al.^55^, H/R was used to injure human umbilical vein endothelial cells (HUVECs), and found that Lnc Neat1 activated the NLRP3 inflammasome by promoting the protein expression levels of BRCA1/BRCA2 Containing Complex Subunit 3 (BRCC3); thus, Lnc Neat1 aggravates the inflammatory response and promotes the injury of HUVECs. Similarly, Zhan et al.^56^ revealed that the expression levels of Lnc Neat1 was upregulated in SD rats with diabetic nephropathy, which was associated with increased NLRP3 inflammasome activation. In systemic lupus erythematosus, the expression levels of Lnc Neat1 is abnormally increased, and knockdown of Lnc Neat1 significantly reduces lipopolysaccharide (LPS)-induced inflammatory cytokine expression^57^. Research has shown that hypoxia-induced activation of the NLRP3 inflammasome and associated inflammatory responses occur in part due to HIF-2a-mediated upregulation of Lnc Neat1^24^. This is a novel pathway. Accordingly, it is curious to speculate that Lnc Neat1 plays a crucial role in MIRI that enables the assembly and activation of NLRP3 inflammasome. Using lentivirus knocked down Lnc Neat1 in HL-1 cardiomyocytes, we additionally demonstrated that Lnc Neat1 enhances the activation of NLRP3 inflammasome thus promotes pyroptosis and inflammatory cascades in vitro (Figs. 6a and 3a). Hence, knocking down Lnc Neat1 in HL-1 cardiomyocytes by using lentivirus, we additionally demonstrated that Lnc Neat1 enhances the activation of NLRP3 inflammasome, thus promotes pyroptosis and inflammatory cascades in vitro (Figs. 6a and 3a). Taken together, these results imply that CGA inhibited pyroptosis and inflammatory response via inhibiting Lnc Neat1/NLRP3 inflammasome, thus protected against MIRI in mice.

In this study, CGA exhibited a significant anti-inflammatory effect in protecting against MIRI, indicating its potential value for clinical application. However, there are challenges that need to be addressed in translating this finding into clinical practice. Firstly, the optimal dosage and administration route of CGA for maximal efficacy and minimal side effects need to be determined. Secondly, further investigation is required to ensure the long-term safety of CGA and to study its potential interactions with other drugs. Finally, large-scale clinical trials are necessary to evaluate the clinical efficacy of CGA in preventing and treating MIRI.

**Figure 7:**
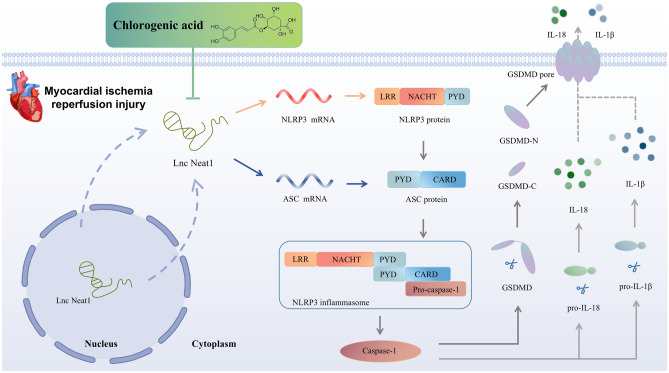
Schematic diagram shows that chlorogenic acid protects against MIRI by inhibiting Lnc Neat1/NLRP3 inflammasome activation-mediated pyroptosis.

In conclusion, the present study provides evidence that CGA may contribute to the protection against MIRI by inhibiting the activation of NLRP3 inflammasome via downregulating Lnc Neat1, which is achieved by alleviating pyroptosis and decreasing inflammatory cascades (Fig. 7). This study may extend a better understanding of the mechanisms underlying CGA protection against MIRI and provide a new strategy for ischemic heart diseases.

### Supplementary Information

Supplementary Figures.

## Data Availability

The datasets generated during and/or analysed during the current study are available from the corresponding authors on reasonable request.
