# Supplementary material for: Chlorogenic acid protects against myocardial ischemia–reperfusion injury in mice by inhibiting Lnc Neat1/NLRP3 inflammasome-mediated pyroptosis
Source: Sci Rep. 2023 Oct 18;13:17803. doi: 10.1038/s41598-023-45017-2 (PMC10584886; doi:10.1038/s41598-023-45017-2)
Supplement: Supplementary file 1 — Supplementary Figures. [file 41598_2023_45017_MOESM1_ESM.docx]

**Chlorogenic acid protects against myocardial ischemia-reperfusion injury in mice by inhibiting Lnc Neat1/NLRP3 inflammasome-mediated pyroptosis**

Xin Cha^1,2,3,+^, Zhengwei Liang^2,3,+^, Junshi Zhang^2,3^, Jing Ding^2,3^, Qian Zhang^2,3^, Sha Lv^2,3^, Yazhu Deng^2,3^, Rongrui Zhang^2,3^, and Deqin Lu^2,3^,*

^1^ The Affiliated Hospital of Guizhou Medical University, Department of Radiology, Guiyang, 550004, China

^2^ Guizhou Medical University, Guizhou Provincial Key Laboratory of Pathogenesis and Drug Research on Common Chronic Diseases, Guiyang, 550004, China

^3^ Guizhou Medical University, Department of Pathophysiology, Guiyang, China

*corresponding.author: [dqlu91@hotmail.com](mailto:dqlu91@hotmail.com)

^+^these authors contributed equally to this work


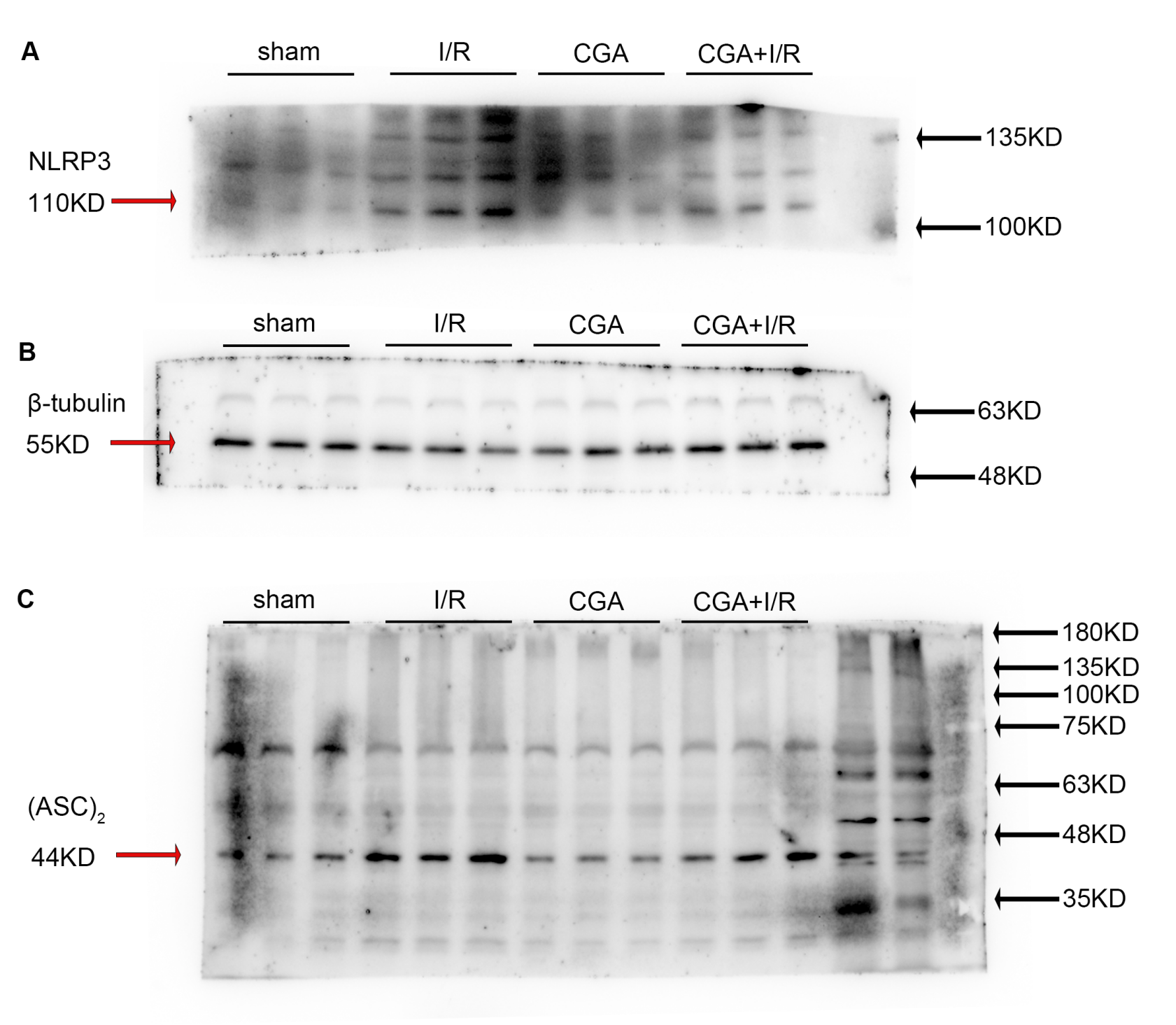


**Supplementary Figure 1 . The full-length blots is the display of cropped blots from Figure 2c and e.**

**Supplementary Figure 2 . The full-length blots is the display of cropped blots from Figure 3c and e.**

**
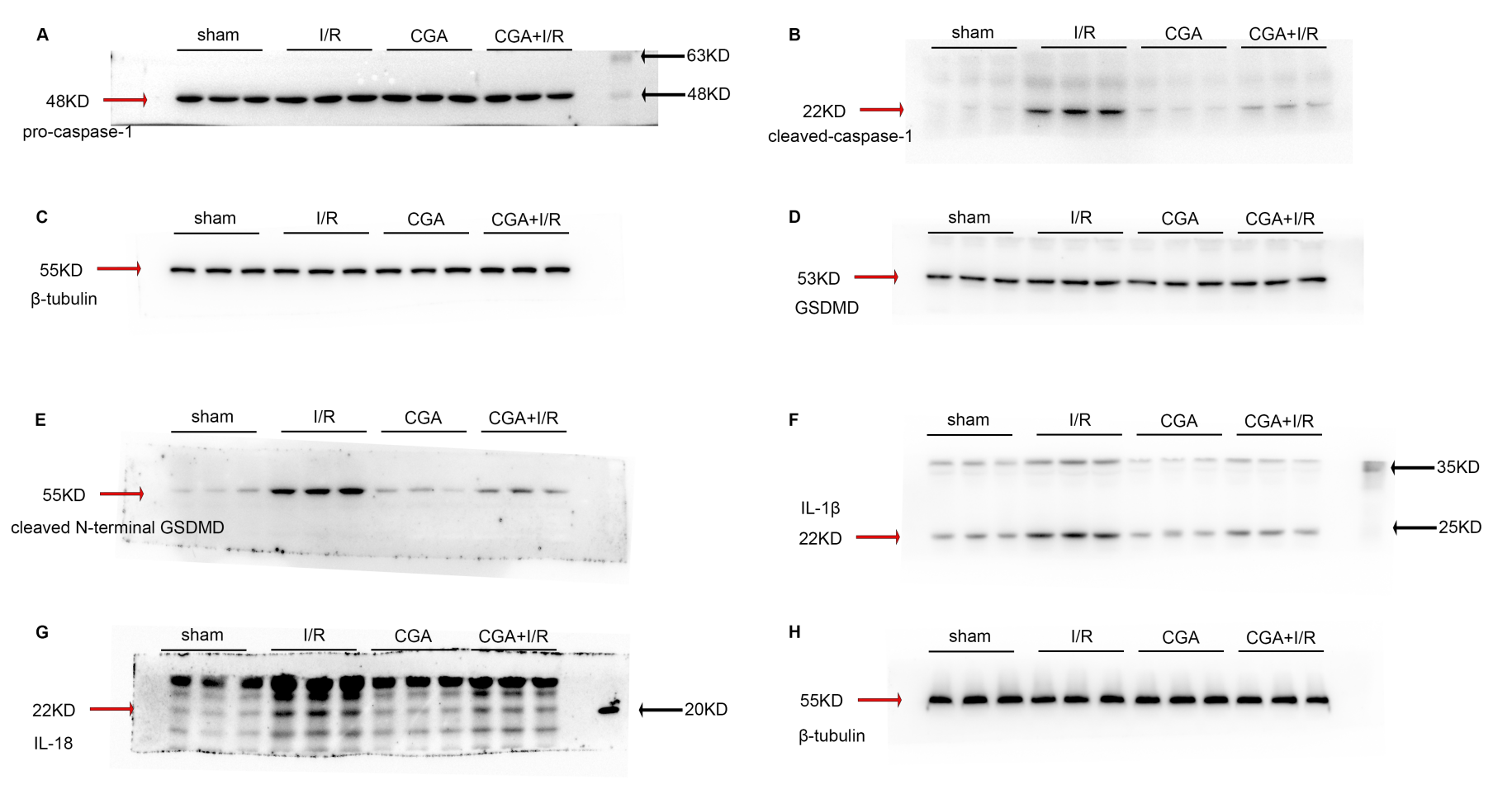
**

**Supplementary Figure 3 . The full-length blots is the display of cropped gels and blots from Figure 4a and c.**

**
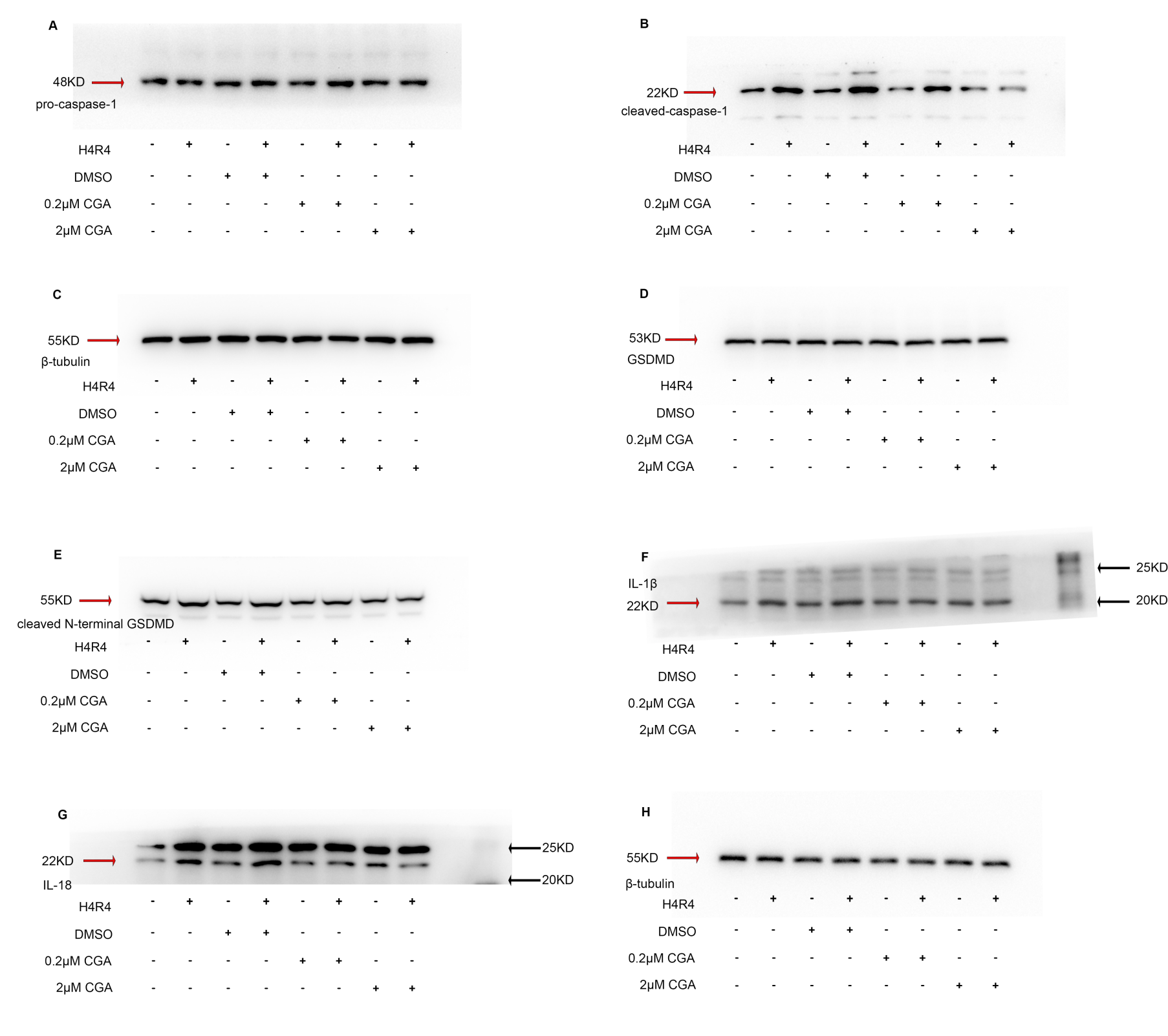
**

**Supplementary Figure 4 . The full-length blots is the display of cropped gels and blots from Figure 5a and c.**


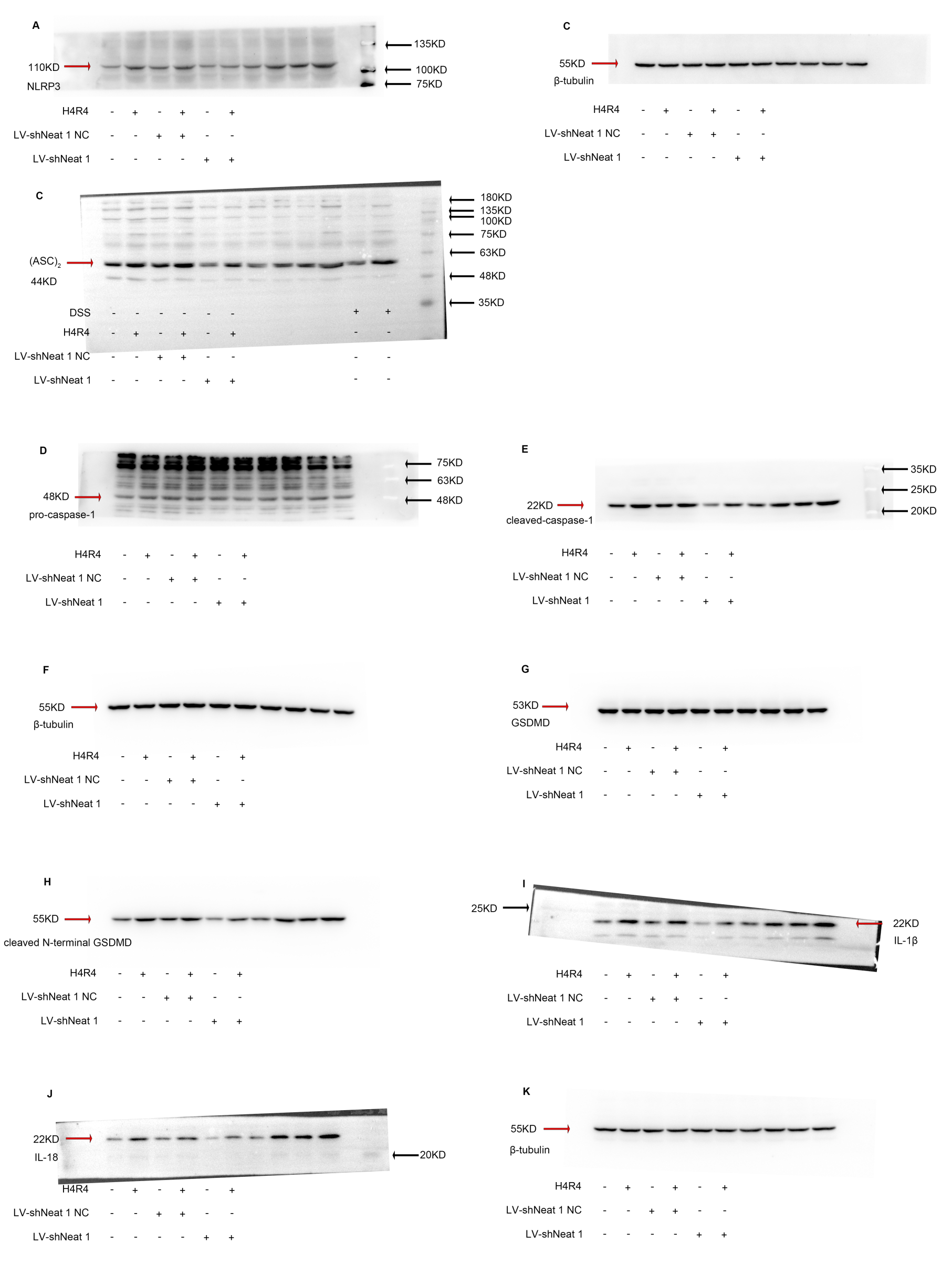


**Supplementary Figure 5 . The full-length blots is the display of cropped gels and blots from Figure 6b, d, e and g.**
